# Supplementary material for: Prevalence estimates of putatively pathogenic leptin variants in the gnomAD database
Source: PLoS One. 2022 Sep 19;17(9):e0266642. doi: 10.1371/journal.pone.0266642 (PMC9484668; doi:10.1371/journal.pone.0266642)
Supplement: S2 Table — This represents the in silico predictions for each variant present in all populations by various tools, namely SIFT [20], PROVEAN [24], PolyPhen2 [21], MutationTaster2021 [22] and FATHMM-MKL [23]. The tools are ordered by their reported accuracy (left: highest accuracy; right: lowest accuracy) [40]. The literature references refer either to a reported clinical case or a functional study. (PDF) [file pone.0266642.s002.pdf]

S2 Table

rsID	Consequence	Homozygous	Heterozygous	*In silico* tools					Described in literature?
				SIFT	PROVEAN	PolyPhen2	MutationTaster 2021	FATHMM-MKL	
rs200092598	p.Gly8Arg	0	4	Tolerated	Neutral	Benign	Benign	Neutral	No
rs1401846669	p.Phe9Leu	0	1	Tolerated	Neutral	Benign	Benign	Damaging	No
rs775874401	p.Leu10Phe	0	6	Tolerated	Neutral	Probably damaging	Benign	Damaging	No
rs767150017	p.Leu12Ile	0	3	Tolerated	Neutral	Possibly damaging	Benign	Damaging	No
rs1036136017	p.Pro14Leu	0	2	Tolerated	Neutral	Benign	Benign	Neutral	No
rs760165439	p.Tyr15Cys	0	2	Tolerated	Neutral	Benign	Benign	Neutral	No
rs765869630	p.Leu16Val	0	1	Tolerated	Neutral	Probably damaging	Benign	Neutral	No
rs148407750	p.Tyr18Cys	0	102	Tolerated	Damaging	Benign	Benign	Neutral	No
rs200179130	p.Gln20Glu	0	1	Tolerated	Neutral	Benign	Benign	Neutral	No
rs1304598562	p.Asp29Gly	0	1	Tolerated	Damaging	Possibly damaging	Benign	Neutral	No
rs752496962	p.Thr33Asn	0	4	Tolerated	Neutral	Probably damaging	Benign	Damaging	No
rs752496962	p.Thr33Ile	0	1	Tolerated	Neutral	Benign	Benign	Neutral	No
rs747703977	p.Ile35del	0	2	In-frame deletion			Benign	In-frame deletion	No
rs111650508	p.Lys36Arg	0	5	Tolerated	Neutral	Possibly damaging	Benign	Damaging	No
rs1342830248	p.Thr40Ile	0	1	Tolerated	Damaging	Benign	Benign	Damaging	No
rs145044661	p.Ile45Val	0	20	Tolerated	Neutral	Benign	Benign	Damaging	No
rs781301976	p.Ile45Asn	0	1	Deleterious	Damaging	Probably damaging	Benign	Damaging	No
rs771590018	p.His47Gln*fs*Ter24	0	2	LoF: high confidence			Deleterious	LoF: high confidence	No
rs770247453	p.Thr48Met	0	4	Tolerated	Neutral	Benign	Benign	Neutral	No
rs770663636	NA	0	1	LoF: high confidence			Deleterious	LoF: high confidence	No
rs776443424	p.Ser52Phe	0	2	Deleterious	Damaging	Probably damaging	Benign	Damaging	No

rs759056593	p.Gln55Lys	0	5	Tolerated	Neutral	Benign	Benign	Damaging	No
rs759056593	p.Gln55Glu	0	1	Tolerated	Neutral	Benign	Benign	Damaging	No
rs200575914	p.Gly59Ser	0	1	Deleterious	Neutral	Probably damaging	Benign	Damaging	Yes [1]
rs751272426	p.Ile63Leu	0	16	Tolerated	Neutral	Benign	Benign	Damaging	No
rs761170909	p.Leu66ArgfsTer51	0	1	LoF: high confidence			Deleterious	LoF: high confidence	No
rs1417571919	p.Ile69Thr	0	3	Tolerated	Neutral	Possibly damaging	Benign	Neutral	No
rs886061973	p.Thr71Asn	0	2	Tolerated	Neutral	Benign	Benign	Neutral	No
rs1332916395	p.Asp76Val	0	1	Deleterious	Damaging	Probably damaging	Deleterious	Damaging	Yes [1]
rs756180416	p.Ala80Ser	0	1	Tolerated	Neutral	Possibly damaging	Benign	Damaging	No
rs780163329	p.Ala80Val	0	1	Tolerated	Damaging	Benign	Benign	Neutral	No
rs1311825614	p.Ile85Phe	0	1	Deleterious	Damaging	Probably damaging	Deleterious	Damaging	No
rs748408158	p.Leu86Phe	0	1	Tolerated	Damaging	Benign	Deleterious	Damaging	No
rs199838573	p.Ser88Cys	0	1	Deleterious	Damaging	Possibly damaging	Benign	Neutral	Yes [1]
rs1231681476	p.Met89Val	0	1	Deleterious	Neutral	Benign	Benign	Neutral	No
rs780990529	p.Pro90Thr	0	1	Deleterious	Damaging	Benign	Benign	Neutral	No
rs17151919	p.Val94Met	105	2167	Tolerated	Neutral	Benign	Benign	Neutral	Yes [2, 3]
rs1226851396	p.Ile95Asn	0	1	Deleterious	Damaging	Probably damaging	Benign	Neutral	No
rs724159998	p.Asp100Asn	0	1	Deleterious	Damaging	Probably damaging	Benign	Damaging	Yes [1, 4]
rs28954113	p.Asn103Lys	0	6	Deleterious	Damaging	Probably damaging	Deleterious	Damaging	Yes [1, 5-10]
rs779227922	p.Leu107Val	0	1	Deleterious	Neutral	Probably damaging	Benign	Damaging	No
rs758826659	p.His109Gln	0	1	Tolerated	Damaging	Benign	Benign	Neutral	No
rs1800564	p.Val110Met	0	43	Tolerated	Neutral	Benign	Benign	Neutral	Yes [11]
rs1800564	p.Val110Leu	0	1	Tolerated	Neutral	Benign	Benign	Neutral	No
rs1211502716	p.Ala112Val	0	1	Deleterious	Damaging	Probably damaging	Benign	Neutral	No
rs1484913857	p.Phe113Leu	0	1	Deleterious	Neutral	Benign	Benign	Neutral	No

rs201114917	p.Lys115Glu	0	1	Deleterious	Neutral	Possibly damaging	Benign	Neutral	No
rs1184373712	p.His118Tyr	0	1	Deleterious	Neutral	Benign	Benign	Neutral	No
rs200202828	p.Ser123Gly	0	4	Tolerated	Neutral	Benign	Benign	Neutral	No
rs1325640667	p.Glu126Gln	0	1	Tolerated	Neutral	Benign	Benign	Neutral	No
rs773031071	p.Thr127Ile	0	2	Deleterious	Damaging	Benign	Benign	Neutral	No
rs760494076	p.Asp129Glu	0	2	Tolerated	Neutral	Benign	Benign	Neutral	No
rs1369849768	p.Ser130Gly	0	1	Tolerated	Neutral	Benign	Benign	Neutral	No
rs1307773933	p.Gly133Val*fs*Ter15	0	7	LoF: high confidence			Deleterious	LoF: high confidence	Yes [12-19]
rs1307773933	p.Val134Cys*fs*Ter38	0	1	LoF: high confidence			Deleterious	LoF: high confidence	No
rs200343690	p.Gly132Arg	0	2	Tolerated	Damaging	Benign	Benign	Neutral	No
rs759465484	p.Gly133Val	0	1	Tolerated	Neutral	Benign	Benign	Neutral	No
rs1478070295	p.Leu135Met	0	1	Tolerated	Neutral	Probably damaging	Benign	Neutral	No
rs759018010	p.Ala137Ser	0	1	Tolerated	Neutral	Benign	Benign	Neutral	No
rs759018010	p.Ala137Thr	0	3	Tolerated	Neutral	Benign	Benign	Neutral	No
rs1392712935	p.Gly139Cys	0	1	Tolerated	Neutral	Benign	Benign	Neutral	No
rs1392712935	p.Gly139Arg	0	1	Tolerated	Neutral	Benign	Benign	Neutral	No
rs367606905	p.Gln151His	0	1	Deleterious	Neutral	Possibly damaging	Benign	Damaging	No
rs757709363	p.Gly152Arg	0	2	Tolerated	Neutral	Benign	Benign	Damaging	No
rs779508377	p.Gly152Glu	0	1	Tolerated	Neutral	Possibly damaging	Benign	Neutral	No
rs754432094	p.Asp156Asn	0	3	Tolerated	Neutral	Benign	Benign	Neutral	No
rs75506045	p.Gln160His	0	4	Tolerated	Neutral	Probably damaging	Benign	Damaging	No
rs191666811	p.Asp162Asn	0	1	Deleterious	Neutral	Probably damaging	Benign	Damaging	No
rs199506740	p.Leu163Arg	0	8	Tolerated	Neutral	Benign	Benign	Neutral	No
rs770761500	p.Ser164Arg	0	1	Tolerated	Neutral	Possibly damaging	Benign	Neutral	No
rs770761500	p.Ser164ThrfsTer30	0	1	LoF: low confidence			Deleterious	LoF: low confidence	No
rs1468690969	p.Pro165Ser	0	1	Tolerated	Neutral	Possibly damaging	Benign	Neutral	No

rs759854910	p.Gly166Arg (G/C)	0	5	Tolerated	Damaging	Possibly damaging	Benign	Neutral	No
rs759854910	p.Gly166Arg (G/A)	0	1	Tolerated	Damaging	Possibly damaging	Benign	Neutral	No
rs1458700374	p.Gly166Ala	0	1	Tolerated	Neutral	Benign	Benign	Neutral	No

References:

1. Bouafi H, Bencheikh S, Mehdi Krami AL, Morjane I, Charoute H, Rouba H, et al. Prediction and Structural Comparison of Deleterious Coding Nonsynonymous Single Nucleotide Polymorphisms (nsSNPs) in Human LEP Gene Associated with Obesity. Biomed Res Int. 2019;2019:1832084.
2. Yaghootkar H, Zhang Y, Spracklen CN, Karaderi T, Huang LO, Bradfield J, et al. Genetic Studies of Leptin Concentrations Implicate Leptin in the Regulation of Early Adiposity. Diabetes. 2020;69(12):2806-18.
3. Friedlander Y, Li G, Fornage M, Williams OD, Lewis CE, Schreiner P, et al. Candidate molecular pathway genes related to appetite regulatory neural network, adipocyte homeostasis and obesity: results from the CARDIA Study. Ann Hum Genet. 2010;74(5):387-98.
4. Dayal D, Seetharaman K, Panigrahi I, Muthuvel B, Agarwal A. Severe Early Onset Obesity due to a Novel Missense Mutation in Exon 3 of the Leptin Gene in an Infant from Northwest India. J Clin Res Pediatr Endocrinol. 2018;10(3):274-8.
5. Wabitsch M, Funcke JB, von Schnurbein J, Denzer F, Lahr G, Mazen I, et al. Severe Early-Onset Obesity Due to Bioinactive Leptin Caused by a p.N103K Mutation in the Leptin Gene. J Clin Endocrinol Metab. 2015;100(9):3227-30.
6. Mazen I, El-Gammal M, Abdel-Hamid M, Amr K. A novel homozygous missense mutation of the leptin gene (N103K) in an obese Egyptian patient. Mol Genet Metab. 2009;97(4):305-8.
7. Shabana, Hasnain S. The p. N103K mutation of leptin (LEP) gene and severe early onset obesity in Pakistan. Biol Res. 2016;49:23.
8. Shabana, Shahid SU, Hasnain S. Identification of genetic basis of obesity and mechanistic link of genes and lipids in Pakistani population. Biosci Rep. 2018;38(4).
9. Wabitsch M, Pridzun L, Ranke M, von Schnurbein J, Moss A, Brandt S, et al. Measurement of immunofunctional leptin to detect and monitor patients with functional leptin deficiency. Eur J Endocrinol. 2017;176(3):315-22.
10. Haglund E, Nguyen L, Schafer NP, Lammert H, Jennings PA, Onuchic JN. Uncovering the molecular mechanisms behind disease-associated leptin variants. J Biol Chem. 2018;293(33):12919-33.
11. Karvonen MK, Pesonen U, Heinonen P, Laakso M, Rissanen A, Naukkarinen H, et al. Identification of new sequence variants in the leptin gene. J Clin Endocrinol Metab. 1998;83(9):3239-42.
12. Farooqi IS. Monogenic human obesity. Front Horm Res. 2008;36:1-11.
13. Farooqi IS, Matarese G, Lord GM, Keogh JM, Lawrence E, Agwu C, et al. Beneficial effects of leptin on obesity, T cell hyporesponsiveness, and neuroendocrine/metabolic dysfunction of human congenital leptin deficiency. J Clin Invest. 2002;110(8):1093-103.

14. Gibson WT, Farooqi IS, Moreau M, DePaoli AM, Lawrence E, O'Rahilly S, et al. Congenital leptin deficiency due to homozygosity for the Delta133G mutation: report of another case and evaluation of response to four years of leptin therapy. J Clin Endocrinol Metab. 2004;89(10):4821-6.
15. Montague CT, Farooqi IS, Whitehead JP, Soos MA, Rau H, Wareham NJ, et al. Congenital leptin deficiency is associated with severe early-onset obesity in humans. Nature. 1997;387(6636):903-8.
16. Fatima W, Shahid A, Imran M, Manzoor J, Hasnain S, Rana S, et al. Leptin deficiency and leptin gene mutations in obese children from Pakistan. Int J Pediatr Obes. 2011;6(5-6):419-27.
17. Saeed S, Bech PR, Hafeez T, Alam R, Falchi M, Ghatei MA, et al. Changes in levels of peripheral hormones controlling appetite are inconsistent with hyperphagia in leptin-deficient subjects. Endocrine. 2014;45(3):401-8.
18. Saeed S, Bonnefond A, Manzoor J, Shabbir F, Ayesha H, Philippe J, et al. Genetic variants in LEP, LEPR, and MC4R explain 30% of severe obesity in children from a consanguineous population. Obesity (Silver Spring). 2015;23(8):1687-95.
19. Saeed S, Butt TA, Anwer M, Arslan M, Froguel P. High prevalence of leptin and melanocortin-4 receptor gene mutations in children with severe obesity from Pakistani consanguineous families. Mol Genet Metab. 2012;106(1):121-6.
