# Supplementary material for: Prevalence estimates of putatively pathogenic leptin variants in the gnomAD database
Source: PLoS One. 2022 Sep 19;17(9):e0266642. doi: 10.1371/journal.pone.0266642 (PMC9484668; doi:10.1371/journal.pone.0266642)
Supplement: S3 Table — This represents all clinical cases reported with either obesity and/or congenital leptin deficiency carrying a LEP variant. Not all reported variants were listed in gnomAD. NA: not available. (PDF) [file pone.0266642.s003.pdf]

S3 Table

Consequence	N	Ethnicity	Gender	Consanguinity	Genotype	In gnomAD?	Reference
c.1-44del42	1	Pakistani	male	Yes	Homozygous	No	[1]
p.Leu12fs	1	Egyptian	female	NA	Homozygous	No	[2]
p.Phe17Leu	1	NA	male	NA	Heterozygous	No	[3]
p.Ile35Ser	1	Egyptian	male	NA	Homozygous	No	[2]
p.Ile35del	1	Pakistani	female	Yes	Homozygous	Yes	[4]
	1	Pakistani	female	Yes	Homozygous		[5]
p.Gln55Stop	1	Indian	female	Yes	Homozygous	No	[6]
p.Leu72Ser	1	Austrian	female	No	Homozygous	No	[7]
p.Asp100Tyr	1	Turkish	male	Yes	Homozygous	No	[8]
p.Asp100Asn	1	Indian	female	Yes	Homozygous	Yes	[9]
p.Asn103Lys	2	German	1 female<br>1 male	No	Homozygous	Yes	[10]
	2	Egyptian	1 female<br>1 male	Yes	Homozygous		[11]
	1	Pakistani	male	Yes	Homozygous		[12]
	1	Pakistani	male	NA	Heterozygous		[13]
p.Arg105Trp	1	Egyptian	female	NA	Homozygous	No	[2]
	3	Turkish	2 female<br>1 male	Yes	Homozygous		[14]
p.Val110Met	1	Finnish	male	NA	Heterozygous	Yes	[15]

p.Cys117Phe	2	Columbian	female	Yes	Homozygous	No	[16]
p.Cys117Tyr	1	Pakistani	male	Yes	Homozygous	No	[1]
p.His118Leu	1	Chinese	NA	NA	Heterozygous	No	[17]
Trp121Stop	2	Egyptian	male	Yes	Homozygous	No	[18]
p.Gly133ValfsTer15	2	Pakistani	1 female<br>1 male	Yes	Homozygous	Yes	[19]
	1	Pakistani	male	Yes	Homozygous		[20]
	1	Pakistani	female	Yes	Homozygous		[21]
	3	Pakistani	NA	NA	Homozygous		[22]
	7	Pakistani	3 female<br>4 male	6 Yes<br>1 No	Homozygous		[5]
	9	Pakistani	4 female<br>5 male	Yes	Homozygous		[4]
	13	Pakistani	8 female<br>5 male	Yes	5 homozygous<br>8 heterozygous		[23]
	11	Pakistani	8 female<br>4 male	Yes	Homozygous		[1]
p.Ser141Cys	4	NA	NA	NA	2 homozygous<br>2 heterozygous	No	[24]
p.Leu161fs17	1	Pakistani	male	Yes	Homozygous	No	[5]
Gross deletion of all coding exons	1	NA	male	Yes	Homozygous	No	[25]

References:

1. Saeed S, Bonnefond A, Manzoor J, Shabbir F, Ayesha H, Philippe J, et al. Genetic variants in LEP, LEPR, and MC4R explain 30% of severe obesity in children from a consanguineous population. Obesity (Silver Spring). 2015;23(8):1687-95.
2. ElSaeed G, Mousa N, El-Mougy F, Hafez M, Khodeera S, Alhelbawy M, et al. Monogenic leptin deficiency in early childhood obesity. Pediatr Obes. 2020;15(1):e12574.
3. Echwald SM, Rasmussen SB, Sorensen TI, Andersen T, Tybjaerg-Hansen A, Clausen JO, et al. Identification of two novel missense mutations in the human OB gene. Int J Obes Relat Metab Disord. 1997;21(4):321-6.
4. Saeed S, Butt TA, Anwer M, Arslan M, Froguel P. High prevalence of leptin and melanocortin-4 receptor gene mutations in children with severe obesity from Pakistani consanguineous families. Mol Genet Metab. 2012;106(1):121-6.
5. Fatima W, Shahid A, Imran M, Manzoor J, Hasnain S, Rana S, et al. Leptin deficiency and leptin gene mutations in obese children from Pakistan. Int J Pediatr Obes. 2011;6(5-6):419-27.
6. Thakur S, Kumar A, Dubey S, Saxena R, Peters AN, Singhal A. A novel mutation of the leptin gene in an Indian patient. Clin Genet. 2014;86(4):391-3.
7. Fischer-Posovszky P, von Schnurbein J, Moepps B, Lahr G, Strauss G, Barth TF, et al. A new missense mutation in the leptin gene causes mild obesity and hypogonadism without affecting T cell responsiveness. J Clin Endocrinol Metab. 2010;95(6):2836-40.
8. Wabitsch M, Funcke JB, Lennerz B, Kuhnle-Krahl U, Lahr G, Debatin KM, et al. Biologically inactive leptin and early-onset extreme obesity. N Engl J Med. 2015;372(1):48-54.
9. Dayal D, Seetharaman K, Panigrahi I, Muthuvel B, Agarwal A. Severe Early Onset Obesity due to a Novel Missense Mutation in Exon 3 of the Leptin Gene in an Infant from Northwest India. J Clin Res Pediatr Endocrinol. 2018;10(3):274-8.
10. Wabitsch M, Funcke JB, von Schnurbein J, Denzer F, Lahr G, Mazen I, et al. Severe Early-Onset Obesity Due to Bioinactive Leptin Caused by a p.N103K Mutation in the Leptin Gene. J Clin Endocrinol Metab. 2015;100(9):3227-30.
11. Mazen I, El-Gammal M, Abdel-Hamid M, Amr K. A novel homozygous missense mutation of the leptin gene (N103K) in an obese Egyptian patient. Mol Genet Metab. 2009;97(4):305-8.
12. Shabana, Hasnain S. The p. N103K mutation of leptin (LEP) gene and severe early onset obesity in Pakistan. Biol Res. 2016;49:23.
13. Shabana, Shahid SU, Hasnain S. Identification of genetic basis of obesity and mechanistic link of genes and lipids in Pakistani population. Biosci Rep. 2018;38(4).
14. Strobel A, Issad T, Camoin L, Ozata M, Strosberg AD. A leptin missense mutation associated with hypogonadism and morbid obesity. Nat Genet. 1998;18(3):213-5.
15. Karvonen MK, Pesonen U, Heinonen P, Laakso M, Rissanen A, Naukkarinen H, et al. Identification of new sequence variants in the leptin gene. J Clin Endocrinol Metab. 1998;83(9):3239-42.
16. Yupanqui-Lozno H, Bastarrachea RA, Yupanqui-Velazco ME, Alvarez-Jaramillo M, Medina-Mendez E, Giraldo-Pena AP, et al. Congenital Leptin Deficiency and Leptin Gene Missense Mutation Found in Two Colombian Sisters with Severe Obesity. Genes (Basel). 2019;10(5).
17. Zhao Y, Hong N, Liu X, Wu B, Tang S, Yang J, et al. A novel mutation in leptin gene is associated with severe obesity in Chinese individuals. Biomed Res Int. 2014;2014:912052.

18. Mazen I, Amr K, Tantawy S, Farooqi IS, El Gammal M. A novel mutation in the leptin gene (W121X) in an Egyptian family. Mol Genet Metab Rep. 2014;1:474-6.
19. Montague CT, Farooqi IS, Whitehead JP, Soos MA, Rau H, Wareham NJ, et al. Congenital leptin deficiency is associated with severe early-onset obesity in humans. Nature. 1997;387(6636):903-8.
20. Farooqi IS, Matarese G, Lord GM, Keogh JM, Lawrence E, Agwu C, et al. Beneficial effects of leptin on obesity, T cell hyporesponsiveness, and neuroendocrine/metabolic dysfunction of human congenital leptin deficiency. J Clin Invest. 2002;110(8):1093-103.
21. Gibson WT, Farooqi IS, Moreau M, DePaoli AM, Lawrence E, O'Rahilly S, et al. Congenital leptin deficiency due to homozygosity for the Delta133G mutation: report of another case and evaluation of response to four years of leptin therapy. J Clin Endocrinol Metab. 2004;89(10):4821-6.
22. Farooqi IS. Monogenic human obesity. Front Horm Res. 2008;36:1-11.
23. Saeed S, Bech PR, Hafeez T, Alam R, Falchi M, Ghatei MA, et al. Changes in levels of peripheral hormones controlling appetite are inconsistent with hyperphagia in leptin-deficient subjects. Endocrine. 2014;45(3):401-8.
24. Chekhranova MK, Karpova SK, Iatsyshina SB, Pankov Iu A. [A new mutation c.422C>G (p.S141C) in homo- and heterozygous forms of the human leptin gene]. Bioorg Khim. 2008;34(6):854-6.
25. Ozsu E, Ceylaner S, Onay H. Early-onset severe obesity due to complete deletion of the leptin gene in a boy. J Pediatr Endocrinol Metab. 2017;30(11):1227-30.
