# Supplementary material for: Prevalence estimates of putatively pathogenic leptin variants in the gnomAD database
Source: PLoS One. 2022 Sep 19;17(9):e0266642. doi: 10.1371/journal.pone.0266642 (PMC9484668; doi:10.1371/journal.pone.0266642)
Supplement: S4 Table — This presents the prevalence estimations using varying definitions of pathogenicity. Here, all variants are classified as ‘pathogenic’ if they were predicted as harmful by at least two in silico tools and were either reported in a clinical case or in a functional study. Further, solely variants listed in the non-synonymous and LoF LEP variants of gnomAD were included in the calculations. NA: not available. (PDF) [file pone.0266642.s004.pdf]

S 4 Table

Population	Pathogenicity definition	Number of carriers of pathogenic variants		Estimated prevalence of heterozygous mutations	Estimated prevalence of homozygous and compound heterozygous mutations
		Heterozygous	Homozygous		
**All populations**	At least *two tools* with a pathogenic prediction or a *case report* or a *functional implication*	2,279	105	1 : 57	1 : 13,000
	At least *two tools* with a pathogenic prediction or a *case report*	112	0	1 : 1,300	1 : 6,380,000
	At least *two tools* with a pathogenic prediction or a *functional implication*	2,234	105	1 : 58	1 : 13,400
**African-American**	At least *two tools* with a pathogenic prediction or a *case report* or a *functional implication*	2,106	104	1 : 6	1 : 120
	At least *two tools* with a pathogenic prediction or a *case report*	7	0	1 : 1,800	1 : 12,730,000
	At least *two tools* with a pathogenic prediction or a *functional implication*	2,106	104	1 : 6	1 : 120
**Ashkenazi Jewish**	At least *two tools* with a pathogenic prediction or a *case report* or a *functional implication*	27	0	1 : 200	1 : 148,000
	At least *two tools* with a pathogenic prediction or a *case report*	0	0	NA	NA
	At least *two tools* with a pathogenic prediction or a *functional implication*	27	0	1 : 200	1 : 148,000

**East-Asian**	At least *two tools* with a pathogenic prediction or a *case report* or a *functional implication*	16	0	1 : 620	1 : 1,560,000
	At least *two tools* with a pathogenic prediction or a *case report*	16	0	1 : 620	1 : 1,560,000
	At least *two tools* with a pathogenic prediction or a *functional implication*	13	0	1 : 770	1 : 2,360,000
**European, Finnish**	At least *two tools* with a pathogenic prediction or a *case report* or a *functional implication*	9	0	1 : 1,400	1 : 7,790,000
	At least *two tools* with a pathogenic prediction or a *case report*	9	0	1 : 1,400	1 : 7,790,000
	At least *two tools* with a pathogenic prediction or a *functional implication*	0	0	NA	NA
**European, non-Finnish**	At least *two tools* with a pathogenic prediction or a *case report* or a *functional implication*	103	0	1 : 630	1 : 1,570,000
	At least *two tools* with a pathogenic prediction or a *case report*	50	0	1 : 1,300	1 : 6,680,000
	At least *two tools* with a pathogenic prediction or a *functional implication*	77	0	1 : 840	1 : 2,820,000
**Latino/Admixed American**	At least *two tools* with a pathogenic prediction or a *case report* or a *functional implication*	169	1	1 : 100	1 : 43,000
	At least *two tools* with a pathogenic prediction or a *case report*	8	0	1 : 2,200	1 : 19,630,000
	At least *two tools* with a pathogenic prediction or a *functional implication*	167	1	1 : 100	1 : 44,000

**Others**[a]	At least *two tools* with a pathogenic prediction or a *case report* or a *functional implication*	35	0	1 : 100	1 : 43,000
	At least *two tools* with a pathogenic prediction or a *case report*	7	0	1 : 520	1 : 1,070,000
	At least *two tools* with a pathogenic prediction or a *functional implication*	33	0	1 : 110	1 : 48,000
**South Asian**	At least *two tools* with a pathogenic prediction or a *case report* or a *functional implication*	24	0	1 : 640	1 : 1,630,000
	At least *two tools* with a pathogenic prediction or a *case report*	15	0	1 : 1,000	1: 4,120,000
	At least *two tools* with a pathogenic prediction or a *functional implication*	21	0	1 : 730	1: 2,130,000
