# Supplementary material for: Prevalence estimates of putatively pathogenic leptin variants in the gnomAD database
Source: PLoS One. 2022 Sep 19;17(9):e0266642. doi: 10.1371/journal.pone.0266642 (PMC9484668; doi:10.1371/journal.pone.0266642)
Supplement: Attachment — Submitted filename: Response to Reviewers.docx [file pone.0266642.s005.docx]

**Response to Reviewer**

***Comments to the Author*** *1. Is the manuscript technically sound, and do the data support the conclusions?

The manuscript must describe a technically sound piece of scientific research with data that supports the conclusions. Experiments must have been conducted rigorously, with appropriate controls, replication, and sample sizes. The conclusions must be drawn appropriately based on the data presented.*

*Reviewer #1: Yes*

**Response: We thank the reviewer for the positive evaluation.**

*2. Has the statistical analysis been performed appropriately and rigorously?*

*Reviewer #1: N/A*

**Response: We have performed the (statistical) analysis appropriately and rigorously. We have even added more analyses and data regarding the leptin gene as a comparable study very recently published (PLoS One) for the FTO gene in weight regulation (Souza Junior et al. 2022 PMID: 34990463).**

*3. Have the authors made all data underlying the findings in their manuscript fully available?

The* [*PLOS Data policy*](http://www.plosone.org/static/policies.action#sharing) *requires authors to make all data underlying the findings described in their manuscript fully available without restriction, with rare exception (please refer to the Data Availability Statement in the manuscript PDF file). The data should be provided as part of the manuscript or its supporting information, or deposited to a public repository. For example, in addition to summary statistics, the data points behind means, medians and variance measures should be available. If there are restrictions on publicly sharing data—e.g. participant privacy or use of data from a third party—those must be specified.*

*Reviewer #1: Yes*

**Response: We again thank the reviewer for the positive evaluation.**

*4. Is the manuscript presented in an intelligible fashion and written in standard English?

PLOS ONE does not copyedit accepted manuscripts, so the language in submitted articles must be clear, correct, and unambiguous. Any typographical or grammatical errors should be corrected at revision, so please note any specific errors here.*

*Reviewer #1: Yes*

**Response: We thank the reviewer.**

*5. Review Comments to the Author

Please use the space provided to explain your answers to the questions above. You may also include additional comments for the author, including concerns about dual publication, research ethics, or publication ethics. (Please upload your review as an attachment if it exceeds 20,000 characters)*

*Reviewer #1: Dear authors,
It was my pleasure to read your manuscript entitled "Prevalence Estimates of Putatively Pathogenic Leptin Variants in the gnomAD Database", but unfortunately I am suggesting the journal to reject it for publication.
My recommendation is based on the fact that it doesn't add anything new and the whole paper is actually based on a very simple calculation. How many potentially pathogenic variants have been identified in the investigated gene, extract the combined minor allele frequency and then by using the Hardy Weinberg equilibrium, to estimate the prevalence of affected individuals.
As I hope you can understand, this calculation while valid (I would suggest though using the ACMG criteria and not merely basing potential pathogenicity on a majority vote among prediction algorithms) and well presented, is not enough for a full research article. If you are interested in pursuing further this type of research I would suggest to concentrate not on one gene, but a disease, correlate with clinical data and draw conclusions about the concordance or discordance between them, while making a mini literature review of the subject.*

**Response:**

**We are grateful for the very positive evaluation and that the manuscript was a pleasurable read. Thus, we hope that other reader would also profit from our manuscript.**

**We like to point out our novel findings. Our analysis adds to the scientific knowledge pertaining to the leptin gene, as we have calculated not only the prevalence of homozygotes and heterozygotes of leptin variants, but also of carriers of compound heterozygous variants. This has never been reported before for leptin variants derived from a public database. We are the first to report frequencies derived from the currently largest database comprising more the 125,000 individuals (Gnomad). We have also calculated population-specific and gender-specific prevalences. These analyses have not been published before.**

**We have also checked a large number of *in silico* tools to obtain educated knowledge about the functional relevance or pathogenicity of the detected variants.**

**Thus, our study adds substantial knowledge to genetic variations in the leptin gene and the implications for obesity in general.**

**To point out that the HWE test is appropriate (as pointed out by the reviewer) we added a respective reference (Lines 128-129): ‘assumption of a perfect population (see Equation (1); p = allele frequency of allele A, q = allele frequency of allele a) as performed by Nunziata et al. (17).**
